# Retaining doctors in organisations in socioeconomically deprived areas in England: a qualitative study

**DOI:** 10.1136/bmjopen-2025-100694

**Published:** 2025-05-08

**Authors:** Liz Brewster, Clare Mumford, Tasneem Patel, Choon Key Chekar, Michael Lambert, Cliff Shelton, Euan Lawson

**Affiliations:** 1 Lancaster Medical School, Lancaster University, Lancaster, UK

**Keywords:** Health Workforce, Health policy, Human resource management

## Abstract

**Abstract:**

**Objectives:**

To identify factors that improve retention in under-doctored areas that experience difficulties in maintaining sufficient medical workforce.

**Design:**

Qualitative study based on semi-structured interviews, collected as part of a larger study.

**Setting:**

Four purposely sampled geographic case study sites in England. Three case study sites were selected as areas that struggled to recruit and retain doctors and one as an area that is oversubscribed. This comprised 27 NHS Trusts, plus 1449 GP practices.

**Participants:**

100 National Health Service (NHS)-employed doctors (including general practitioners, consultant specialists, specialty and specialist doctors, resident doctors/doctors in postgraduate training and locally employed doctors) were interviewed between December 2022 and March 2024.

**Findings:**

Participants shared their experiences of organisational levers that impact on decisions about working life and retention in the workforce. Two key themes explained factors influencing retention. First, participants discussed feeling valued by the organisation, both in terms of material circumstances and in relationships with colleagues. Second, the theme of autonomy and opportunity explored why doctors chose to stay in areas that typically experience difficulties in maintaining sufficient staffing.

**Conclusions:**

Many studies focusing on workforce examine why staff leave, but by focusing on factors that influence retention, greater understanding of specific facets of organisational culture can be used to inform policy and practice.

**Trial registration number:**

ISRCTN95452848.

**STRENGTHS AND LIMITATIONS OF THIS STUDY**

- A large and diverse number of doctors participated in the study, purposively sampled to ensure inclusivity.
- Data were collected across multiple organisations, reinforcing the transferability of findings.
- Participants were self-selecting, which can be considered a limitation as it is difficult to know how widely representative their experiences are.
- We did not collect data from doctors who had left medical work, which may have provided further insights into workforce retention.
- Data were collected as part of a wider study on medical training and careers, meaning some opportunities to drill down into detail of retention issues may have been missed.

## Introduction

 Providing healthcare services that meet the needs of the population—universal health coverage—relies on having sufficient medical workforce to provide those services.^1^ In the UK, as internationally, there is widely acknowledged to be a healthcare workforce ‘crisis.’^2^ Numeric estimates of workforce shortages vary, but note that the UK has higher vacancy rates and lower average numbers of doctors per 100 000 population than comparable countries, a shortfall in general practitioners (GPs) and unfilled long-term/permanent positions that are then covered by higher-cost short-term locum doctors.^3^,^5^ This shortfall has implications for patient care, as well as the cost of service provision. Shortages of healthcare professionals persist over time, and interventions remain limited, often focusing on government action on providing and/or subsidising more education and training places to grow the workforce.^6^

Workforce distribution is a geographical problem, with fewer doctors in primary and secondary care in some areas, despite the greater healthcare needs of the population.^7^ In England, recent analyses have demonstrated that the most deprived areas have 1.4 fewer full-time equivalent GPs per 10 000 population than the least deprived, and similar patterns of deprivation affecting distribution are also seen in other countries including Canada and Australia.^8^,^10^ Not attracting enough primary and secondary care medical professionals to work in an area affects the lived experience of patients and their health outcomes, including unequal distribution of avoidable mortality.^11^,^13^

Efforts to solve the global healthcare workforce crisis focus on two areas: recruitment and retention. Retaining staff has been identified as a priority area in the UK and internationally.^2 14 15^ Evidence suggests that more senior and experienced doctors have a positive impact on efficient and effective medical decision-making and quality of care, and medical leadership is particularly important in relation to mentoring and training future generations of doctors.^16 17^

Studies often focus on factors affecting attrition, including burnout, rather than examining what encourages medical professionals to continue working.^18^,^20^ This gap was noted in a recent review of hospital doctor turnover which highlighted how ‘a lack of focus on doctors who remain in their job hinders a comprehensive understanding of the issue.’^21^ Examining what makes doctors leave is important, but does not address important questions around motivation to remain or improvements to workplaces or job design that could be made. The decision to stay is an ongoing negotiation, and therefore we would argue that attention to the everyday experience of work—what makes it bearable even in difficult circumstances—is vital to avoiding an individual making a one-off decision to leave. Previous research has, in part, undermined attempts to really understand what drives decision-making around staying or leaving by asking about future intentions to leave or stay, rather than focusing on experiences of staying.^22 23^

To address this gap, this study investigates the retention of the medical workforce in England, focusing particularly on areas that are known to have localised issues with recruitment and retention. The paper moves beyond an analysis that prioritises organisational impacts (eg, cost, turnover) to consider a more person-centred notion of what it means to remain in an organisation.

Recent research on retention in specific contexts of medical work supports the need for a more holistic view. In examining the day-to-day working experiences of emergency medicine doctors, one study identified practical solutions that are employed by these doctors to enable them to continue to work in a difficult environment.^24^ This study is notably influential in supporting the re-conceptualisation of notions of retention in terms of actions focused on career sustainability.^24 25^ Research on retention in remote and rural medicine, guided by geographic approaches to migration and rural studies, emphasises how there are diverse influential factors, including sense of belonging and community links, and access to amenities such as schooling and housing, that need to be considered to usefully impact on geographic workforce distribution.^26^,^28^

This study aims to identify factors that positively influence retention in areas that experience difficulties in maintaining a medical workforce. Using data gathered as part of a broader study aiming to understand the influence of medical training pathways on workforce distribution, socioeconomic deprivation and health inequalities, this study focuses on doctors’ experiences of working in an organisation, concentrating on what makes them stay and examining work-related organisational factors. Given the richness of the dataset and the challenges in representing these experiences in appropriate depth, the role of life-related factors influencing retention will be discussed elsewhere.^29^ The research question for this paper was: ‘What organisational factors influence doctors working in areas that struggle to recruit and retain a workforce, that make them want to stay in their current role and/or organisation?’

## Methods

A qualitative approach was selected in order to describe participants’ educational and career pathways and relate them to understanding of workforce data, with a particular focus on what encouraged people to remain working in an organisation over time. Interviews enabled the collection of detailed data about doctors’ working lives, career trajectories and factors influencing their decision making, led by the individual doctors and prompted by open questions (online supplemental file 1). Data were collected as part of a wider study, results of which are presented elsewhere.^30^ Analysis was conducted reflexively, using a data-driven approach.^31^ Our epistemological stance was broadly social constructionist, emphasising the role of interchanges and exchanges in creating a shared understanding of the world, with reference to individual histories, biographies and positionality.^32^ Our large and diverse sample of doctors, alongside our rigorous and robust analysis process, ensures the transferability and relevance of findings to other settings.

### Recruitment and participants

We recruited 100 participants from four geographic case study sites, which comprised 27 NHS organisations, plus 1449 GP practices (table 1). Case studies were selected as areas that struggled to recruit and retain doctors in three cases and one area that has been consistently oversubscribed. Case boundaries were defined based on regional NHS structures (eg, Foundation School and Postgraduate Deanery) and refined to focus on inclusion of organisations offering clinical placements to selected medical schools, as per the broader research questions for the wider study.

**Table 1:** Overview of case studies, medical schools, GP practices and NHS Trusts

Case study site	Medical school(s)	Number of GP practices*	Number of NHS Trusts
North West	Lancaster Medical School, University of Central Lancashire Medical School	195	4
Northern and North East	Newcastle Medical School, University of Sunderland Medical School	363	10
Lincolnshire	Lincoln Medical School	80	3
North London (oversubscribed site)	Imperial College School of Medicine, UCL Medical School, Barts and The London School of Medicine and Dentistry	811	10

* Taken from NHS Digital *Data for General Medical Practices, General Medical Practitioners, Prescribing Cost Centres and Dispensaries, supplied by the NHS Prescription Services (NHS PS*) uploaded 30 August 2024 and mapped to case study area boundaries.

GP, general practitioner; NHS, National Health Service.

We identified eligible doctors working in the case studies via an open invitation to participate, which was distributed via email or in organisational newsletters, and with the support of local research infrastructure (NIHR Clinical Research Networks). Organisations supported the research by sharing recruitment materials with all medical staff but played no further role in the study.

All participants were medically qualified and were employed in a variety of clinically active roles. All doctors working within case study sites were eligible to participate, and the sample of 100 doctors, approximately 30 from each site which struggled with recruitment/retention, and 10 from the oversubscribed site, was regularly reviewed with an aim to purposively sample to try to ensure inclusivity (including across primary/secondary care; considering age, gender, disability, socioeconomic background, ethnicity and career stage). Sample size was assessed using the concept of information power to ensure that the research questions for the overall study, including its broad aim and scope for cross-case study analysis, could be answered appropriately.^33^

### Data collection

Semistructured interviews based on an interview schedule (online supplemental file 1) were used to collect data. They broadly followed a narrative chronological structure guiding the participant through their career to date, supplemented by some reflective questions on recruitment and retention, and the purpose of medical education. Interview questions explored decision-making, motivations and priorities, with an awareness of the structures that organise medical training in the UK context. Interviews were conducted by a health psychology researcher (TP), a medical educator and medical sociologist (LB) and a sociologist (CKC). Interview recruitment commenced in December 2022 in the first case study, with rolling case study recruitment until all interviews were complete by March 2024. Interviews typically lasted 1 hour and were conducted online or via telephone, depending on participant availability. In-person interviews were offered, but all participants preferred the convenience of online/telephone conversation. All interviews were audio-recorded and fully transcribed by a professional transcriber.

### Patient, public and stakeholder involvement

Patients and the public were involved prior to, and throughout the study. Patient groups informed the research questions as part of the study design, and a patient and public involvement and engagement (PPIE) group of eight individuals met regularly throughout the research study to provide feedback on emerging findings, share their experiences and concerns about healthcare provision and access to services. Their insights emphasised the value they placed on continuity of care, concerns about malpractice and priorities for service provision. These insights were integrated into the interpretation of study data, for example, the analysis focused on retention presented in this study speaks directly to PPIE interest in seeing the same doctor/continuity of care.

Alongside PPIE, we also included input from doctors. Two members of the research team are registered doctors (a GP and a consultant anaesthetist) who provided input into the research design, including pilot testing the interview schedule. A medical careers advisory group of diverse professionals at different career stages also provided reflections on the findings.

### Ethical issues

Ethical approval was granted by Lancaster University FHM Research Ethics Committee in August 2022. Health Research Authority approval was granted in September 2022, and participating organisations completed a non-commercial Organisation Information Document to confirm capacity and capability to support the research. Written informed consent was given to the research team by all participants. Given the potential for detailed career narratives to be identifiable, all data extracts presented here have been anonymised, reported using minimal identifiers (eg, participant number, role, location), and demographic data aggregated.

### Data analysis

Data analysis was conducted using a data-driven constant comparison approach that uses conceptual ordering to develop theory.^34^ It foregrounded experiences that have shaped pathways through medical training, understanding key moments of change and identifying considerations that influence decisions about retention, or continuing to work in an organisation versus leaving for another role. Data were managed in Atlas.ti 24 and Atlas.ti Web to facilitate secure collaborative analysis with large datasets.

Participants were recruited from across NHS organisations in case study sites but, due to the organisation of medical education and training in the UK, had worked in more than one organisation (inside or outside the case study boundaries), meaning they were able to comment on a wide range of working environments and reflect on moments of change such as moving organisation. The analysis process involved several phases, conducted concurrently with data collection. First, LB and TP worked with the first 10 interview transcripts to create a preliminary coding framework, via data immersion through creating detailed summaries of the transcripts and then looking across them for commonalities and differences. TP then applied this preliminary framework to the first 20 transcripts and collated interview extracts. Team discussion between LB, CKC and CM, an organisational work and technology researcher, refined this framework which was then applied to all transcripts. Preliminary themes were then generated through interrogation of the coding framework, conducted by CM supported by LB and CKC.

Throughout this process, themes were refined and solidified around consideration of ‘push’ and ‘pull’ factors that prompted decision-making around staying or leaving an organisation or an area. As per our focus on retention, we prioritise discussion of ‘pull’ factors in the following section. These factors were contrasted with wider participant narratives, which reflected on what participants saw as key considerations of career or wider life, and how decision-making was usually multidimensional and not driven by one discrete concern. Analytical concepts were discussed with the wider team, including an NHS historian (ML), a consultant anaesthetist (CS) and a GP (EL). By integrating analysis in this way, we were able to create an explanatory account of working lives that moves beyond describing individual career pathways to examine systems of workforce distribution that affect retention and start to account for the previously identified differences in retention rates across organisations.

## Findings

### Participant characteristics

Data were collected from interviews with 100 doctors. All participants provided demographic data (table 2); we were able to recruit a diverse sample of doctors, including a good mix of gender, age, ethnicity, role and specialism, including primary and secondary care and length of working life. Although participants were diverse, the key themes identified were visible across narratives of very different doctors, showing the transferability of findings.

**Table 2:** Interview participant demographics

Current role	Doctor in postgraduate training (resident doctor)	30
	General practitioner	42
	Specialty and specialist doctor or locally employed doctor	7
	Consultant specialist	21
Primary Medical Qualification region	UK	80
	International	20
Gender	Female	49
	Male	48
	Other gender identity/not recorded	3
Age range	21–24	1
	25–34	31
	35–44	34
	45–54	24
	55–64	10
Ethnicity	Asian or Asian British	21
	Black or Black British	9
	Mixed	3
	White	61
	Other	5
	Not recorded	1
Case study region/short name	Lincolnshire/Lincs	30
	Northern and North East/NE	29
	North West/NW	31
	North London/Lon	10

### Overview of key themes

Participants shared their experiences of organisational levers or tangible elements of policy and process that organisations could attend to, in order to improve the working lives of their employees. These organisational levers impacted decisions about working life, and two key themes explained factors influencing retention. Aspects of organisational culture that influence retention were clearly visible.

First, participants discussed feeling valued by the organisation, both in terms of material circumstances and in relationships with colleagues. Second, the theme of autonomy and opportunity explored why doctors chose to stay in areas that typically experience difficulties in maintaining sufficient staffing. This feeling of autonomy was particularly noticeable in relation to identifying future opportunities or potential pathways for them. These opportunities could be related to their ability to take on particular roles or responsibilities, or a perception that they were able to make a greater difference to patient care and outcomes. However, experiences varied over time, with several of those who had more recently joined the profession and/or were still training commenting that they could see a shift towards having less autonomy and control.

Factors such as quality of life and family responsibilities need to be acknowledged as influential in decision-making, but are not discussed here as the study aims to contribute to discussion of potential interventions or changes that could be directly enacted by healthcare organisations. These organisational factors influenced decision-making when participants were reflecting on whether an organisation presented a positive working environment, and ultimately influenced their decision to stay.

### The importance of feeling valued in retention

The idea of feeling valued by an organisation was discussed by participants across diverse organisations and job roles. As an example, one GP questioned the relationship between the idea of being valued by an organisation and the framing of organisational priorities around recruitment and retention.

Retention: what does that mean? Does it mean having someone in a job forever, even though they’re miserable? Is it retaining them for a year, is it retaining them for 5 years? I wouldn’t use the word retention. I think I would say “nurture and sustain”: that’s what I would use. You don’t retain your kids, do you? You nurture and you sustain them and support them. (P023, GP, NW)

This reframing of the terminology around retention spoke to the greater conceptualisation of ‘value’. Value was not just about financial recompense for tasks, it was a deeper and more meaningful commitment from an employer. Other participants operationalised this commitment in terms of being given time and support. A resident doctor discussed why they wanted to stay in the place they were currently training, citing two examples of what made them feel valued.

Everybody pulls their weight, so it makes it easy to work there. The two trainers are committed to training. They don’t negotiate with you about tutorial times: tutorial time is tutorial time. […] if somebody puts on your list a problem patient, somebody will send you a message and say, “Make sure you’ve really looked this patient up, if you want to have a chat about them before you see them or after you’ve seen them, I’m here”. You know, it’s amazing. So I find that it’s a place that I think I would thrive. (P020, resident doctor, Lincs)

This commitment to time for training and support for management of complex patients was seen as positive aspects of the culture, focused on paying attention to nurturing doctors from an early stage. Alongside this ‘softer’ value, others identified very practical and material things that organisations could do to support their staff to make them feel valued, accounting for their day-to-day needs.

What is attractive are packages, basic stuff like having a car park where people can park their car in hospital; having a canteen where you can get food after seven. The hospital doesn’t stop at seven o’clock in the evening, there are doctors, nurses, staff all the time, 24/7, they haven’t got a place to eat. […] Unless one can do those small things […] it will be the same uphill struggle to recruit doctors. (P038, consultant specialist, NW)

While those settled in open-ended roles or established careers reflected on what encouraged them to stay in an organisation, others who were earlier in their careers and still on the training pathway considered what might encourage ongoing engagement. Reflections from those earlier in their career are particularly pertinent when thinking about retaining the workforce of the future. In the UK, resident doctors have short-term appointments and move around and work in different organisations as part of their training process. This movement leads to doctors recognising the differences between places and potentially influences future decision-making. A resident doctor summarised these differences in discussion of ‘added extras’.

It’s not just monetarily…it’s the little things. For example, at [organisation A], they would give you like a Christmas hamper box thing and £100 bonus, which isn’t a huge amount of money but it meant a lot, and that made a big difference to people’s morale. I remember everybody was a lot more jolly around then. Compare that to [organisation B], I think they gave us something like a 50 quid [£50] bonus, but nothing else, and then at [organisation C], we got nothing whatsoever, and at [organisation D], we got nothing whatsoever…It just felt like they were making an effort, whereas in these other places, you think you’re just one of the numbers, really. (P090, resident doctor, NE)

This direct comparison between organisations emphasised the significance of organisational culture for participants and accentuated the implications of feeling valued for retention.

### Autonomy and opportunity for doctors enable retention

Alongside strongly expressed views about feeling valued, another frequently occurring theme centred on the idea of doctors having autonomy and control and how the presence or perception of opportunities enabled retention.

Many of those interviewed had stayed in one location for a long period of time. One consultant specialist, who had worked in the same organisation for over 20 years, spoke about how he had been given autonomy and was able to craft his own job design to facilitate job satisfaction and how this was vital for keeping him in the role; again, he compared his current place of work with his previous one.

This place was more a Yes place where [location A] is a No place. So I came up here and I went, ‘I want to set this up.’ ‘OK, what do you need?’ I got given it, and I went, ‘Oh, can I do some of this?’ They were like: ‘Yes, what else would you want?’ So there was opportunity, and autonomy and opportunity were the things that kept me in the job for as long as I did. (P064, consultant specialist, NW)

A desire for autonomy also affected the specialisms and roles that doctors chose, when faced with consideration of taking on responsibilities within a healthcare system.

Wherever I’ve worked, I’ve always been a partner. I don’t want to be a salaried GP, I don’t want to be told what to do. (P035, GP, NW)

This prioritisation of autonomy is particularly relevant in relation to considerations of wider organisational structures. As our case studies centred on areas that struggle to recruit and retain, many of the areas discussed were not ones that would typically be seen as prestigious. These smaller, less prestigious sites did not offer opportunities that might typically be seen as attractive, such as being a tertiary or specialist centre, having a strong research reputation or being an internationally recognisable brand. However, participants found these environments provided greater opportunity for autonomy and potential to develop further skills.

Participants identified these benefits when they spoke about having greater control over rotations and pathways and knowing patients and systems. GPs, consultant specialists, locally employed doctors, and specialty and specialist doctors, who were often more embedded in place by virtue of having a longer-term position (in contrast with a rotational training position), all spoke about the opportunities of smaller places.

For one GP who had trained and was now working in a more remote and rural area, the benefits of working in a smaller regional system were clear. He was able to shape and secure the training placements he wanted and to work closely with more senior clinicians who were role models. This culminated in taking on an extended role, enabling him to pursue a special interest alongside his main role as a GP. The characteristics of the region that some saw as a disadvantage, in terms of being more remote and less prestigious, actually provided the conditions in which he was able to take on greater responsibility, which led to high job satisfaction. This ability to adapt and take on a wider portfolio of work led him to describe how he had ‘found a nice niche for me to exist in’ (P002, GP, Lincs)*.* Higher job satisfaction was also visible in relation to provision of patient care and working relationships.

I’ve enjoyed that more intimate feeling that you get working in a smaller place. And being able to make a bigger difference, perhaps, to your population in a smaller hospital than you feel that you do [in a place] when there’s lots of egos! (P037, consultant specialist, NW)

While this consultant specialist was reflecting back on his career to date, similar sentiments were also visible in comments by more recent medical graduates, who were looking forward to future opportunities.

I see [place] as a growing trust […] and especially with the new medical school, I see a lot of opportunities for someone who is just growing their career, rather than working in the a trust that is already made. So I would rather stay in a growing trust where I am sure I have good prospects of career growth, than working in a big trust that is already made and I may be lost even within the trust. (P013, SAS doctor, Lincs)

Considering these examples, it is clear that protective factors that support retention include job satisfaction and being given autonomy and opportunity but also broader infrastructure, including a good working environment.

## Discussion

The findings from our qualitative study, presented here, outline work-related factors associated with retention of the workforce in areas that struggle to recruit and retain. Retention is as important as recruitment to ensure workforce sustainability. We have centred doctors’ experiences of everyday work to illustrate key concerns and now turn to identify how these experiences may inform change at a system level. Our large-scale study considers what factors may be influenced at an organisational level to improve working lives for doctors, as well as what factors may influence the decision to stay in an area that broadly struggles to retain doctors. The findings highlight that while these areas may experience some disadvantages in terms of material resources, location and reputation, those who work in these areas could still see benefits of remaining in the workforce in these organisations. We refer to these as ‘organisational levers’ or tangible elements of policy and process that organisations could attend to, in order to improve the working lives of their employees and thus encourage them to continue to work in their organisation. First, doctors were more likely to remain in role if they felt valued by the organisation. This was both in terms of resources and renumeration, but more widely in relation to the support for their education, training and well-being. Second, whether doctors felt that they had autonomy over their working lives, and opportunities afforded by this autonomy, was also a driver for retention.

Strengths of this study are that we included a large sample of doctors, purposively sampled and reflexively reviewed to ensure inclusion of a wide range of characteristics. By using the concept of information power, we are confident that we collected rich data allowing us to answer our research questions.^33^ However, we are aware that with such a large sample, our representation of the multiple subjectivities within the data can only ever be partial. Limitations include that participants were self-selecting and that we have only engaged with those who currently work in the NHS in our case study sites, meaning that we are not collecting insights from those who have left the geographic areas we are working in or the health service entirely. Previous research, which did include those who had left a specialty as well as those remaining in it, found that similar experiences could be identified across those who had stayed and those who had left, so we are confident that our findings are representative more widely.^24^ Data were collected as part of a wider study on medical training and careers, meaning some opportunities to drill down into detail of retention issues may have been missed.

Findings presented here contribute significantly to understandings of the retention of the medical workforce with a view to intervention and improvement through identifying relevant modifiable factors. High-quality evidence on the topic of retention has previously been identified as a gap in the literature.^35^ A recent systematic review on turnover and retention specifically calls for qualitative studies to support moves towards a deeper understanding of the topic.^21^ Despite considerable attention being paid to the topic, much research focuses on identifying factors leading to burnout and attrition rather than trying to understand what encourages doctors to stay.^18 36^ Other relevant studies frame the contribution of their findings around well-being, which—while important—positions the outcome as focused on improving circumstances for the individual doctor, rather than centring the role of healthcare service providers.^37^ Our findings in part align with one of the most influential reports on well-being, *Caring for Doctors, Caring for Patients,* which also addresses the importance of autonomy for doctors.^38^ Perhaps surprisingly, given the relevance of the findings for healthcare leadership, line managers and leaders were rarely explicitly discussed in these interviews as barriers or enablers to retention.

The findings here also make a further contribution to knowledge by asking doctors to review their careers retrospectively. Previous research aiming to identify what influences doctors’ decisions around factors such as location and specialty relies on more quantitative methods including discrete choice experiments.^39^,^41^ This methodology looks prospectively at what doctors identify as important in their decision-making. Our research instead focused in depth on what had happened in doctors’ careers, which was often different from where doctors thought their career path would take them. This enabled us to think about what factors sustained this work over time.

The implications of this study are relevant internationally as well as to UK healthcare policymakers and managers. While we have focused here on organisational level factors and not engaged with wider determinants driving retention, we have identified several modifiable factors that could be better accounted for when considering working lives in healthcare settings. These factors align with similar findings from Ireland, where listening and responding to staff concerns and meeting core needs at work were seen as integral to job satisfaction, and thus retention.^42 43^ These organisational factors demonstrate the potential of organisations to drive change and to consider how to enable the retention of staff in areas that may face shortfalls in staffing. Many of the barriers to retention are practical and align with previously identified factors associated with attrition or staff turnover.^21^ However, by focusing on retention, and on both those who had long-lived careers and those who were earlier in their career journey, we present an account that will contribute to improving future healthcare service provision.

When comparing those earlier in their careers to those later in working life, we noted that change over time was visible, both in terms of what was prioritised and when it was prioritised. Understanding these complex, nuanced accounts of medical careers as experienced by doctors is relevant for workforce planning. These accounts also have implications for medical leadership; many of those who were more senior in their careers held leadership roles, and they were shaped by their experiences of what it meant to be in a role with greater autonomy and sense of opportunity earlier in their career. Overall, this study identifies work-related organisational factors, which may form the basis of practical recommendations for healthcare managers and policy-makers.

## Supplementary material

10.1136/bmjopen-2025-100694 online supplemental file 1

## Data Availability

Data are available upon reasonable request.
