# Supplementary material for: Retaining doctors in organisations in socioeconomically deprived areas in England: a qualitative study
Source: BMJ Open. 2025 May 8;15(5):e100694. doi: 10.1136/bmjopen-2025-100694 (PMC12067819; doi:10.1136/bmjopen-2025-100694)
Supplement: online supplemental file 1 [file bmjopen-15-5-s001.docx]

**Supplementary file: Interview Question topic guide**

**Welcome and introduction - Seek consent to continue, reminder of recording of interview, re-cap of project and plan for interview.**

1. Can you tell me what your current role is?

Prompts: How long have you worked in that role?

1. Where did you grow up/ go to school?

Prompts: What kind of area was that? When you think back, what do you associate with that place? Do you still have family/ links with [place]? Have any other members of your family trained as doctors?

1. Where did you go to medical school?

Prompts: When/ what year was that? How did you end up at [place] school? What influenced your choice and/or decision? What did you think of it at the time? When you think back, what do you associate with that place? Which hospitals did you go to on placements? What specialties were you exposed to on your placements? What was your view on those placements? Do you still have links with [place]? Were there other things on your mind at the time? (further prompts – specialism in medicine, life events, family illness, meeting partner, having fun, children etc – to develop as appropriate, led by participant).

1. Where was your first post-medical school training placement?

Prompts: When/ what year was that? Can you talk me through how you selected that placement? What did you think of it at the time? When you think back, what do you associate with that place? Do you still have links with [place]? Were there other things on your mind at the time? (further prompts – specialism in medicine, life events, family illness, meeting partner, having fun, children etc – to develop as appropriate, led by participant).

**[depending on career stage]** *Using same questions, prompt discussion around second placement, training programme, academic fellowships, completion of college exams, further place-based impressions and decision making until get back to current role.*

1. We often hear that workforce recruitment and retention are a problem in [location]. Is this something that you identify with as a narrative?

Prompts: Do you think it’s a popular location to come for training? (why/ why not?) Does it attract graduates from a particular medical school? Is this the case for all hospitals or specialisms?

1. Can you reflect on what the goal is of medical education in the UK when you trained?

Prompts: How far has it been achieved? Is it the same today? What is the aim, in terms of types of future doctors? Has this been constant or changed over time?

**Anything not covered?**

**Closing -** Thank for their time and contribution.
