# Supplementary material for: Retaining doctors in organisations in socioeconomically deprived areas in England: a qualitative study
Source: BMJ Open. 2025 May 8;15(5):e100694. doi: 10.1136/bmjopen-2025-100694 (PMC12067819; doi:10.1136/bmjopen-2025-100694)
Supplement: Supplementary file 2 [file bmjopen-2025-100694.reviewer_comments.pdf]

PEER REVIEW HISTORY

BMJ Open publishes all reviews undertaken for accepted manuscripts. Reviewers are asked to complete a checklist review form and are provided with free text boxes to elaborate on their assessment. These free text comments are reproduced below.

ARTICLE DETAILS

Title (Provisional)

Retaining doctors in organisations in socio-economically deprived areas in England: a qualitative study

Authors

Brewster, Liz; Mumford, Clare; Patel, Tasneem; Chekar, Choon Key; Lambert, Michael; Shelton, Cliff; Lawson, Euan

---

VERSION 1 - REVIEW

**Reviewer**	**1**
**Name**	**Voss, Sarah**
**Affiliation**	**University of the West of England, Health and Life Sciences**
**Date**	**13-Mar-2025**
**COI**	**None**

This is a well-presented paper that describes a qualitative investigation of the factors affecting retention of UK doctors in deprived areas. It is clear and well-written and of interest to a wider readership. I have only a couple of minor points for the authors to consider.

a) This data was collected as part of a larger study and this is made clear in the manuscript. However, I think there are a couple of opportunities to make this clearer. I think this could be included in the abstract and perhaps also discussed as a potential limitation. Looking at the topic guide, it seems there is only one question targeted to the aim of this particular study and if the data was collected as part of a wider interview agenda, there may have been missed opportunities to drill into the detail of retention issues.

b) Is it possible to include data on length of service or length of time in role of Table 2? The authors mention that many of those interviewed has stayed in one location for along period of time. It would be helpful to quantify this and perhaps include a sentence that describes the sample in the context of the wider population.

c) In the discussion, there is an opportunity to draw on some theories of occupational psychology e.g. social exchange theory

Reviewer **2**
Name **Moussallem, Marianne**
Affiliation **Saint Joseph University of Beirut, Higher Institute of Public Health**
Date **15-Mar-2025**
COI **None**

The article presents an interesting study that tackles an important topic. The authors selected a relevant study design, with well-described methods. Further contextualization and additional comparisons with existing literature would significantly improve the quality of the discussion and provide better orientation for readers who may not be very familiar with the context. Detailed comments for improvement are outlined below:

Title:

• It would be beneficial to specify the country in which the study was conducted. Socio-economically deprived areas in England are very different from those in other countries.

Introduction:

• Page 3, Line 11: Consider comparing with other benchmarks for a more comprehensive introduction.

• Page 3, Lines 37-45: This section could be expanded to provide a clearer discussion of the factors at play.

Methodology:

• Page 4, Lines 3-4: "Factors influencing their decision-making" – Could you be more specific regarding the nature of these factors, especially as factors related to personal life are not considered?

• Under "Patient and Public Involvement": Could you please provide more concrete details on how this involvement influenced the design, analysis, and interpretation of findings?

• Under "Ethical Approval": "Written informed consent was given by all participants." Was this consent provided by the doctors to their organizations? Additionally, it might be useful to mention potential bias, given that recruitment was conducted through the organization, which could influence the narratives of participants.

• Under "Data Analysis," Lines 39-43: It would be helpful to include the idea of participants having worked in more than one organization under the "Recruitment and Participants" section. This is an important factor for determining eligibility for participation.

Discussion:

• The "Strengths and Limitations" section would be better placed at the end of the discussion.

• Page 11, First Paragraph: This section would benefit from additional explanation and more concrete examples from the literature that align with your findings.

By incorporating these suggestions, the article can achieve greater clarity and depth, ultimately improving its contribution to the field. I appreciate the effort put into this study and look forward to seeing the revised version.

**Reviewer**	**3**
**Name**	**Burford, Bryan**
**Affiliation**	**Newcastle University, School of Medicine**
**Date**	**22-Mar-2025**
**COI**	**None**

Comments to authors

This is an important and timely piece of work given the ongoing challenges of recruitment and retention in the UK medical workforce.

Overall, the paper is a well-written account of a well-executed study. Numbered suggestions for revision below are intended to offer improvements to allow further development some interesting findings. I have noted some typos, but probably not all, and a careful proof read is recommended. Some sections/paras feel a bit dense, and I also suggest a further pass of precis to thin things out. That will release some words for expansion of the findings section.

Introduction

1. Typo first sentence – meets not meet

2. Suggest reconsidering the sentence ‘This maldistribution means that the international crisis around medical workforce is also a localised issue, which has significantly higher impacts in some regions compared to others’. It is perhaps unnecessary in context anyway, but carries a perhaps unintended implication that workforce issues locally could be seen as an expression of a global issue. While global economic, political and environmental contexts do have an effect on individual healthcare systems, it seems more likely that the reverse is true – that localised issues may find parallels elsewhere, leading to an apparent global problem.

3. Typo c line 21 – unnecessary ‘the’ before ‘senior’.

4. The sentence ‘A further complication is that many previous studies trying to understand staff retention have concentrated on intention to stay or leave rather than the experience of staying or leaving’ makes an important point that could perhaps be teased out a little bit. A

focus on precursors to exit is intuitive, if the intention of research is to avoid exit, and so a more detailed explanation of why it is important to understand those who did not make that decision would help. The following para goes some way to this, but I felt the point could be more emphatic.

There's also a perhaps more abstract question about how continuous a process that from intention to decision is. That is, those who are currently 'retained' may still have an intention, or ideation, of leaving – exit is a finite and discrete decision, but staying is not. Put another way, someone only has to decide to leave once, but they decide to stay every day. I think this could be developed more throughout – the seed is in this para, but it does not seem to germinate.

5. While the final para describes a broad aim, research questions are not stated, despite being referred to elsewhere in the paper. Clear RQs would help illustrate the overall coherence and symmetry of the paper.

Methods

6. The 'recruitment and participants' section doesn't report the number of participants- while the information is in the findings section, it seems an omission here in the context of talking about a purposive sample and target sample size.

7. This paper represents a very large qualitative dataset. While the metatheoretical framing, and broad methodological approach, is appropriate, I would have liked to see some comment on the challenges of negotiating such a large sample, and whether there is an epistemological tension in seeking to combine multiple subjectivities. The concept of 'information power' I felt needed more explanation and justification. To my reading it is epistemologically problematic in the context of a constructionist approach – it seems to present 'information' as a finite resource to be tapped from participants, rather than knowledge being something which is generated within the research process. The size and breadth of the sample does not really feel like it pays off in the findings, and so more consideration of methodological challenges, and epistemological considerations, would help.

It's a perennial problem when trying to ensure representation across areas and demographics that not everything fits into a results section, but some acknowledgement of that, and whether it was addressed, would be welcome. (Not necessarily just in the methods, although it is a challenge within analysis).

8. As well as PPIE, was there a medical professional stakeholder group providing input to sampling and interview design?

9. The analysis describes identification of push and pull factors, but the findings seem to focus on pull factors. I recognise the value of this, but a more explicit statement of that intent, and of justification of that approach would have smoothed the transition from methods to findings. (Was awareness of appreciative enquiry part of this?)

Findings

10. I felt the sample characteristics would be better described first, and then followed by the introduction to the themes.

11. The first two paras I felt could be simplified, and shortened, to set up the main description of the themes, allowing the themes themselves to present more depth and detail.

The focus on the positives, and the ongoing attractors which keep people in their roles, that I'd expected from the intro was not really foregrounded. Allowing the themes more room to breathe may have helped this.

12. I can see the argument against including 'holistic' factors (although I felt there may be a better term to reflect these being personal things, outside of work), but also wondered about the interaction between these things and experience of work (bearing in mind the influence that such factors have on recruitment and choice of job). The justification of their exclusion may need another look, and I think a note in the discussion.

13. The use of 'intrinsic' evoked 'intrinsic motivation' for me – I'm not sure if that's a deliberate echo, but while autonomy seemed to reflect that sense, some of the features mentioned under 'feeling valued' seemed quite extrinsic: 'packages, basic stuff like having a car park where people can park their car in hospital; having a canteen where you can get food after seven'. The argument that these things are symbolic, rather than simply transactional, is apparent, and I'm perhaps being picky about language, but it jarred a little.

14. An incidental thought – 'what might encourage them to remain – or be retained, from an organisational perspective' captures a further tension in the question of retention, which may have been present in data. 'Remaining' suggests active agency, whereas 'being retained' places agency with the organisation, and could suggest the doctor is a more passive participant in their employment. I wondered if this was apparent in how participants presented their decisions? Consider a comment on this in the discussion, even if additional data does not fit into the findings.

'Feeling valued' section

15. Sentence 'While those settled in permanent careers' might be better worded as 'established careers' – 'permanent' has implications about their commitment and retention that may not be true, or intended.

16. The final para containing 'not on other factors such as geographic location or wider quality of life' makes assumptions about intra-regional homogeneity which I don't think stand up. Within each of the under-doctored regions, there are differences in geography and demography which can mean a very different style and quality of life. The main point about organisational culture is well made, but I don't think the implication of region being a control for geography supports the argument.

Autonomy and opportunity section

17. I felt autonomy was a bit buried in this section, and if cuts are made elsewhere, this will benefit most from expansion. The first example seems to reflect being valued, rather than having control. It is opportunity, but directly given rather than afforded by context. More interpretation here may make the link to autonomy clearer, but overall the section could do with more evidence of the importance of autonomy. (I think a link to the GMC's 'Caring for doctors Caring for patients' report would be appropriate in the discussion to pick up the importance of autonomy).

18. p8 Garbled sentence: 'many of the locations that those working in these areas were not ones that would typically seen as prestigious'. This and following para could be simplified – I think the main point. that smaller, less clinically prestigious sites may afford greater autonomy and opportunity, is also a bit buried and could be reworked more simply.

Discussion

19. The beginning of the discussion inverts the findings somewhat, from the participants experience to their providing 'organisational levers'. I think that's a useful inversion, and provides a route from individual understanding to potential system change, but it felt a bit abrupt – a sentence or two acknowledging and steering that change of viewpoint would have been more convincing.

20. While I appreciate the argument for 'informational power', and putting aside my scepticism as to its value, I do think the size of the sample is a limitation when it comes to synthesising multiple viewpoints. Any inter-regional differences (and indeed interpersonal differences) within the sample aren't really reflected in the findings as reported. That's as likely to be a limitation of the form (i.e. a fairly short paper) as of the methods, but with a large qual sample I always wonder about the stories unexplored by the need to cover a larger volume of data.

21. Typo 'we have only engaging'

22. The discussion doesn't always reflect (my reading of) the findings. For example, 'Our research found that quite often, what doctors thought would be important was overshadowed by other concerns in their lived experience of decision-making throughout their careers.' This Is an interesting observation, but I don't think it was readily apparent in the findings. Similarly, 'we have identified several modifiable factors' doesn't reflect my reading of the findings. Enumerating them in the discussion would reinforce any echo of the data, but I would suggest making sure that any such statements in the discussion are clearly foreshadowed in the framing of the data and examples provided.

VERSION 1 - AUTHOR RESPONSE

Reviewer: 1
Prof. Sarah Voss, University of the West of England
Comments to the Author:
This is a well-presented paper that describes a qualitative investigation of the factors

affecting retention of UK doctors in deprived areas. It is clear and well-written and of interest to a wider readership. I have only a couple of minor points for the authors to consider.

Thank you for these positive comments

a) This data was collected as part of a larger study and this is made clear in the manuscript. However, I think there are a couple of opportunities to make this clearer. I think this could be included in the abstract and perhaps also discussed as a potential limitation. Looking at the topic guide, it seems there is only one question targeted to the aim of this particular study and if the data was collected as part of a wider interview agenda, there may have been missed opportunities to drill into the detail of retention issues.

We have added references to the wider study, strengths and limitations and discussion, as suggested.

While there was only one question focused on retention in a geographical area in the abstract sense, much of the interviews covered the participant's personal journey through their medical career, including multiple points of staying or leaving in different organisations, meaning we were able to discuss experiences of being retained and decision making experiences throughout the interviews. Most of the quotes given as examples, and analytical work completed focused on the interviews as a whole giving many opportunities for probing questions e.g. 'what made you stay in X organisation at that time?' The career narrative interview approach built rapport with participants, leading to detailed reflections on staying and leaving, led by participants not pre-existing hypotheses about what might contribute to retention.

b) Is it possible to include data on length of service or length of time in role of Table 2? The authors mention that many of those interviewed has stayed in one location for along period of time. It would be helpful to quantify this and perhaps include a sentence that describes the sample in the context of the wider population.

We did not systematically collect data on length of service and so are not able to add this to table 2. We are not aware of any data generally on length of service in one organisation at the individual level, as reckonable service in the NHS tends to be counted continuously (e.g. length of time working for any NHS organisation) rather than length of time working for one organisation.

c) In the discussion, there is an opportunity to draw on some theories of occupational psychology e.g. social exchange theory

While we appreciate that social exchange theory might be a useful theorisation, we are not occupational psychologists by background. Having reviewed the literature on this topic, the concept itself seems to be criticised for its limited theoretical precision, and so we are unwilling to add this theorisation in here, partly given the range of ways in which it is applied, and partly because there is not scope within the article to develop these points in full given the limitations of the wordcount. We are happy to take a steer from the editor if it is felt that this is something that should be included.

Reviewer: 2
Dr. Marianne Moussallem, Saint Joseph University of Beirut

Comments to the Author:
The article presents an interesting study that tackles an important topic. The authors selected a relevant study design, with well-described methods. Further contextualization and additional comparisons with existing literature would significantly improve the quality of the discussion and provide better orientation for readers who may not be very familiar with the context.

Thank you for these positive comments.

Detailed comments for improvement are outlined below:
Title:
• It would be beneficial to specify the country in which the study was conducted. Socio-economically deprived areas in England are very different from those in other countries.

This has been changed as suggested.

Introduction:
• Page 3, Line 11: Consider comparing with other benchmarks for a more comprehensive introduction.

We have added references to similar patterns of workforce distribution in Australia and Canada.

• Page 3, Lines 37-45: This section could be expanded to provide a clearer discussion of the factors at play.

We have expanded this section to state a few relevant factors identified, including sense of belonging and community links, and, access to facilities such as schooling and housing.

Methodology:
• Page 4, Lines 3-4: "Factors influencing their decision-making" – Could you be more specific regarding the nature of these factors, especially as factors related to personal life are not considered?

While we do not directly report factors relating to personal life in this paper, interviews covered the intersection between work life and personal life. As per our exploratory approach, the interviews were led by the individual doctors and their life stories, prompted by open questions. We have added this as a clarification, with the suggestion that the interview schedule as supplementary file supports this. Given the diversity of career stages and roles, our question set needed to be open to understanding what might have influenced decisions, so we used prompts as appropriate (e.g. further prompts – specialism in medicine, life events, family illness, meeting partner, having fun, children etc – to develop as appropriate, led by participant).

• Under "Patient and Public Involvement": Could you please provide more concrete details on how this involvement influenced the design, analysis, and interpretation of findings?

We have revised this section to clarify how PPIE group insights informed interpretation of findings, linking their interest in continuity of care with this paper's theme of retention.

• Under "Ethical Approval": "Written informed consent was given by all participants." Was this consent provided by the doctors to their organizations? Additionally, it might be useful to mention potential bias, given that recruitment was conducted through the organization, which could influence the narratives of participants.

Doctors were contacted through their organisations and volunteered to participate independently, without this being reported to their organisations, minimising the potential for bias. It was made clear in recruitment materials that this was an independent research project, not commissioned by the organisations the doctors worked for. Doctors organisations then had no role in the consent process. A line has been added to clarify this in the article.

• Under "Data Analysis," Lines 39-43: It would be helpful to include the idea of participants having worked in more than one organization under the "Recruitment and Participants" section. This is an important factor for determining eligibility for participation.

The only eligibility criteria was currently working in one of the NHS organisations selected for recruitment, and having worked in more than one organisation was not an eligibility criteria. However, because of the design of medical education and training in the UK, it is almost inevitable that a doctor has studied or worked in more than one organisation, and they naturally compared experiences across multiple organisations. A clarification to this effect has been added.

Discussion:
• The "Strengths and Limitations" section would be better placed at the end of the discussion.

We were guided by the BMJ Open guidance on writing a discussion section, which suggests the discussion section should contain "a statement of the principal findings; strengths and weaknesses of the study; strengths and weaknesses in relation to other studies, discussing important differences in results; the meaning of the study: possible explanations and implications for clinicians and policymakers; and unanswered questions and future research."

We therefore propose to leave the strengths and limitations in its current position within the discussion.

• Page 11, First Paragraph: This section would benefit from additional explanation and more concrete examples from the literature that align with your findings.

This paragraph has been revised with suggestions from the literature as suggested.

By incorporating these suggestions, the article can achieve greater clarity and depth, ultimately improving its contribution to the field. I appreciate the effort put into this study and look forward to seeing the revised version.

Reviewer: 3
Dr. Bryan Burford, Newcastle University
Comments to the Author:
Comments to authors
This is an important and timely piece of work given the ongoing challenges of recruitment and retention in the UK medical workforce.
Overall, the paper is a well-written account of a well-executed study.

Thank you for these positive comments

Numbered suggestions for revision below are intended to offer improvements to allow further development some interesting findings. I have noted some typos, but probably not all, and a careful proof read is recommended. Some sections/paras feel a bit dense, and I also suggest a further pass of precis to thin things out. That will release some words for expansion of the findings section.

Thank you for these detailed comments – we appreciate the careful attention paid to our work.

Introduction
1. Typo first sentence – meets not meet

corrected

2. Suggest reconsidering the sentence 'This maldistribution means that the international crisis around medical workforce is also a localised issue, which has significantly higher impacts in some regions compared to others'. It is perhaps unnecessary in context anyway, but carries a perhaps unintended implication that workforce issues locally could be seen as an expression of a global issue. While global economic, political and environmental contexts do have an effect on individual healthcare systems, it seems more likely that the reverse is true – that localised issues may find parallels elsewhere, leading to an apparent global problem.

This sentence was revised as suggested, then removed in further editing, given the suggestion from the reviewer that it was perhaps unnecessary in context.

3. Typo c line 21 – unnecessary 'the' before 'senior'.

corrected

4. The sentence 'A further complication is that many previous studies trying to understand staff retention have concentrated on intention to stay or leave rather than the experience of staying or leaving' makes an important point that could perhaps be teased out a little bit. A focus on precursors to exit is intuitive, if the intention of research is to avoid exit, and so a more detailed explanation of why it is important to understand those who did not make that decision would help. The following para goes some way to this, but I felt the point could be more emphatic.

There's also a perhaps more abstract question about how continuous a process that from intention to decision is. That is, those who are currently 'retained' may still have an intention, or ideation, of leaving – exit is a finite and discrete decision, but staying is not. Put another

way, someone only has to decide to leave once, but they decide to stay every day. I think this could be developed more throughout – the seed is in this para, but it does not seem to germinate.

Changes have been made to these two paragraphs to try and more clearly articulate the importance of focusing on retention, and the ongoing reinforcement of the issue of staying.

5. While the final para describes a broad aim, research questions are not stated, despite being referred to elsewhere in the paper. Clear RQs would help illustrate the overall coherence and symmetry of the paper.

A research question has been added as suggested. The research question for this paper was: 'what organisational factors influence doctors working in areas that struggle to recruit and retain a workforce, that make them want to stay in their current role and organisation?'

Methods
6. The 'recruitment and participants' section doesn't report the number of participants- while the information is in the findings section, it seems an omission here in the context of talking about a purposive sample and target sample size.

The number of participants has been added here as suggested.

7. This paper represents a very large qualitative dataset. While the metatheoretical framing, and broad methodological approach, is appropriate, I would have liked to see some comment on the challenges of negotiating such a large sample, and whether there is an epistemological tension in seeking to combine multiple subjectivities. The concept of 'information power' I felt needed more explanation and justification. To my reading it is epistemologically problematic in the context of a constructionist approach – it seems to present 'information' as a finite resource to be tapped from participants, rather than knowledge being something which is generated within the research process. The size and breadth of the sample does not really feel like it pays off in the findings, and so more consideration of methodological challenges, and epistemological considerations, would help.

It's a perennial problem when trying to ensure representation across areas and demographics that not everything fits into a results section, but some acknowledgement of that, and whether it was addressed, would be welcome. (Not necessarily just in the methods, although it is a challenge within analysis).

Information power is a well established, yet (we would argue) possibly misnamed way to think about qualitative sampling. We prefer it as an explanatory description of what we have done to 'data saturation' as it enables consideration of 'enough' experiences to answer the research questions, without implying we have captured all examples of the phenomenon. We agree with the reviewer that information is not a finite resource.

Malthud et al's original framing of information power states that:

"Our model is not intended as a checklist to calculate N but is meant as a recommendation of what to consider systematically about recruitment at different steps of the research process."

Looking across our broad aim for the study as a whole (not just this sub-analysis focused on retention) it is clear that Malthud et al's modelling work enabled us to articulate and appraise how many participants would enable us to answer our broad research question for the whole study (What factors influence medical students and doctors' decisions about career specialism and geographic location?)

The very large dataset was collected for the whole study, and so we have clarified this in the description of the use of information power. Our overall research aims included cross-case analysis, meaning that 30 participants per site (and 10 in the oversubscribed site) is more in line with established norms of qualitative research when taken at the case level.

We have added a line to the strengths and weaknesses in the discussion to note that we are aware that with such a large sample, our representation of the multiple subjectivities within the data can only ever be partial.

8. As well as PPIE, was there a medical professional stakeholder group providing input to sampling and interview design?

Yes - we have added the following to clarify:

Two of the research team are currently registered doctors (one primary care, one secondary care) who provided input into the research design, including pilot testing the interview schedule. A medical careers advisory group of diverse professionals at different career stages also provided reflections on the findings.

9. The analysis describes identification of push and pull factors, but the findings seem to focus on pull factors. I recognise the value of this, but a more explicit statement of that intent, and of justification of that approach would have smoothed the transition from methods to findings. (Was awareness of appreciative enquiry part of this?)

A sentence has been added to smooth this as suggested: As per our focus on retention, we prioritise discussion of 'pull' factors in the following section.

While we are aware of appreciative enquiry, it was not an explicit framing for this study.

Findings

10. I felt the sample characteristics would be better described first, and then followed by the introduction to the themes.

We have switched these sections round as suggested.

11. The first two paras I felt could be simplified, and shortened, to set up the main description of the themes, allowing the themes themselves to present more depth and detail.

The focus on the positives, and the ongoing attractors which keep people in their roles, that I'd expected from the intro was not really foregrounded. Allowing the themes more room to breathe may have helped this.

These paragraphs have been shortened, and made more focused as suggested.

12. I can see the argument against including ‘holistic’ factors (although I felt there may be a better term to reflect these being personal things, outside of work), but also wondered about the interaction between these things and experience of work (bearing in mind the influence that such factors have on recruitment and choice of job). The justification of their exclusion may need another look, and I think a note in the discussion.

We have removed the term ‘holistic’ here. We have tried to better justify exclusion of factors outside work in the introduction, clarifying that “Given the richness of the dataset, and the challenges in representing these experiences in appropriate depth, he role of life-related factors to retention will be discussed elsewhere.[29]”

We have added a reference to the paper we have submitted elsewhere which covers this topic, and are happy to provide the submitted version to the BMJ Open editor if helpful to demonstrate that there is no overlap between these two papers.

13. The use of ‘intrinsic’ evoked ‘intrinsic motivation’ for me – I’m not sure if that’s a deliberate echo, but while autonomy seemed to reflect that sense, some of the features mentioned under ‘feeling valued’ seemed quite extrinsic: ‘packages, basic stuff like having a car park where people can park their car in hospital; having a canteen where you can get food after seven’. The argument that these things are symbolic, rather than simply transactional, is apparent, and I’m perhaps being picky about language, but it jarred a little.

We have reviewed, and removed intrinsic/ extrinsic from description of the themes.

14. An incidental thought – ‘what might encourage them to remain – or be retained, from an organisational perspective’ captures a further tension in the question of retention, which may have been present in data. ‘Remaining’ suggests active agency, whereas ‘being retained’ places agency with the organisation, and could suggest the doctor is a more passive participant in their employment. I wondered if this was apparent in how participants presented their decisions? Consider a comment on this in the discussion, even if additional data does not fit into the findings.

While we see what is meant here, we feel it is not what was intended when we used this language to describe the issue. We have clarified to refer to remaining within an organisation as we feel that both the doctor and the organisation have active agency. We are also aware, within our data (and have written about previously published work e.g. https://doi.org/10.1111/1467-9566.13480) that sometimes participants, when asked to tell the story of their career, construct a post-hoc rationalisation of their decision making, which is framed in terms of active agency when this may not have been as clear cut as described (e.g. they work in the only organisation that offered them a job rather than actively choosing the organisation, then post-hoc identify factors that made them choose that organisation).

‘Feeling valued’ section

15. Sentence ‘While those settled in permanent careers’ might be better worded as ‘established careers’ – ‘permanent’ has implications about their commitment and retention that may not be true, or intended.

We have changed as suggested. We were trying to differentiate between those in a role with no end date, and those still on the training pathway, who took short-term roles for six months/ a year and so moving on from the role was part of the process. We have also clarified this.

16. The final para containing 'not on other factors such as geographic location or wider quality of life' makes assumptions about intra-regional homogeneity which I don't think stand up. Within each of the under-doctored regions, there are differences in geography and demography which can mean a very different style and quality of life. The main point about organisational culture is well made, but I don't think the implication of region being a control for geography supports the argument.

This paragraph aims to say that when a trainee was committed to a region, as this trainee was, they were still choosing between the multiple organisations within that region, and it was at this point that organisational culture really had an influence. However, we recognise that there is not scope to detail this or develop the argument in this paper, so we have edited accordingly to try and make this clearer.

Autonomy and opportunity section
17. I felt autonomy was a bit buried in this section, and if cuts are made elsewhere, this will benefit most from expansion. The first example seems to reflect being valued, rather than having control. It is opportunity, but directly given rather than afforded by context. More interpretation here may make the link to autonomy clearer, but overall the section could do with more evidence of the importance of autonomy. (I think a link to the GMC's 'Caring for doctors Caring for patients' report would be appropriate in the discussion to pick up the importance of autonomy).

We disagree here, as the participant themselves frames the example in relation to autonomy and crafting their job design. We have added a clarification here. We have added a reference to Caring for doctors Caring for patients in the discussion as suggested.

18. p8 Garbled sentence: 'many of the locations that those working in these areas were not ones that would typically seen as prestigious'. This and following para could be simplified – I think the main point. that smaller, less clinically prestigious sites may afford greater autonomy and opportunity, is also a bit buried and could be reworked more simply.

This sentence has been simplified and reworked as suggested.

Discussion
19. The beginning of the discussion inverts the findings somewhat, from the participants experience to their providing 'organisational levers'. I think that's a useful inversion, and provides a route from individual understanding to potential system change, but it felt a bit abrupt – a sentence or two acknowledging and steering that change of viewpoint would have been more convincing.

We have added a sentence as suggested.

20. While I appreciate the argument for 'informational power', and putting aside my scepticism as to its value, I do think the size of the sample is a limitation when it comes to synthesising multiple viewpoints. Any inter-regional differences (and indeed interpersonal differences) within the sample aren't really reflected in the findings as reported. That's as likely to be a limitation of the form (i.e. a fairly short paper) as of the methods, but with a

large qual sample I always wonder about the stories unexplored by the need to cover a larger volume of data.

We have engaged with discussion of information power above, and hope the reviewer is reassured by our clarification. We agree that it is a significant challenge to fully represent diverse views in a short paper, but we think the findings are of interest and relevance to the journal audience. This is why we have focused here on one element of the research analysis, workplace culture and retention, rather than trying to present the research study as a whole. We feel that the examples chosen in these accounts would be recognisable to the majority of our participants, even if their experience is not directly represented.

21. Typo ‘we have only engaging’

Corrected

22. The discussion doesn’t always reflect (my reading of) the findings. For example, ‘Our research found that quite often, what doctors thought would be important was overshadowed by other concerns in their lived experience of decision-making throughout their careers.’ This Is an interesting observation, but I don’t think it was readily apparent in the findings. Similarly, ‘we have identified several modifiable factors’ doesn’t reflect my reading of the findings. Enumerating them in the discussion would reinforce any echo of the data, but I would suggest making sure that any such statements in the discussion are clearly foreshadowed in the framing of the data and examples provided.

Our point re prospective versus retrospective methodologies looking at this problem has been clarified. we have tried to focus this on the idea of sustaining a career. In clarification to other reviewers, we have added to the discussion on modifiable factors, so we hope this is now clearer.

VERSION 2 - REVIEW

Reviewer **2**

Name **Moussallem, Marianne**

Affiliation **Saint Joseph University of Beirut, Higher Institute of Public Health**

Date **24-Apr-2025**

COI

Thank you to the authors for addressing the comments and providing the necessary clarifications. I have no further comments and am satisfied with the version shared.
